# Associating Liver Partition with Portal Vein Ligation and Staged Hepatectomy (ALPPS): Feasibility of Performing in Infants with Large Hepatic Tumor—Case Report

**DOI:** 10.3390/healthcare13050460

**Published:** 2025-02-21

**Authors:** Aleksandar Sretenovic, Srdjan Nikolic, Nada Krstovski, Nenad Zdujic, Milan Slavkovic, Ivana Dasic, Dejan Nikolic

**Affiliations:** 1 Department of Pediatric Surgery, University Children’s Hospital, Tirsova 10, 11000 Belgrade, Serbia; aleksandar.sretenovic@udk.bg.ac.rs (A.S.); milan.slavkovic@udk.bg.ac.rs (M.S.); 2 Faculty of Medicine, University of Belgrade, Dr Subotica 8, 11000 Belgrade, Serbia; onkosurge1@yahoo.com (S.N.); nada.krstovski@udk.bg.ac.rs (N.K.); dejan.nikolic@udk.bg.ac.rs (D.N.); 3 Institute for Oncology and Radiology of Serbia, Pasterova 14, 11000 Belgrade, Serbia; 4 Department of Pediatric Oncology, University Children’s Hospital, Tirsova 10, 11000 Belgrade, Serbia; 5 Department of Radiology, University Children’s Hospital, Tirsova 10, 11000 Belgrade, Serbia; ivana.dasic@udk.bg.ac.rs; 6 Department of Physical Medicine and Rehabilitation, University Children’s Hospital, Tirsova 10, 11000 Belgrade, Serbia

**Keywords:** rhabdoid liver tumor, liver resection, hepatobiliary surgery, infant

## Abstract

**Background**: Surgical resection remains an important treatment of choice for a large number of liver tumors in children. Sometimes, if a tumor infiltrates a large part of the liver, after resection, the future liver remnant (FLR) is not enough for normal liver function. The size of the FLR is one of the determining factors for resectability as postoperative liver failure (PLF) is the most severe complication after partial hepatectomy. A new strategy for treating marginally resectable liver tumors in adult patients which were initially considered as unresectable was formally reported in 2011. This operative technique is a hepatectomy consisting of two stages with initial portal vein ligation and in situ splitting of the liver parenchyma. In 2012, the acronym “ALPPS” (associating liver partition and portal vein ligation for staged hepatectomy) was proposed for this novel technique. However, there is a small number of ALPPS procedures performed in pediatric patients published in the literature. **Objectives**: The aim of this paper is to present the first case of a pediatric patient with a marginally resectable rhabdoid tumor of the liver which was initially considered unresectable and who was treated with two-stage hepatectomy. We report a case of a 4-month-old girl with a large rhabdoid tumor of the liver who underwent this procedure. **Conclusions**: ALPPS can be a valuable technique to achieve complete resection of pediatric liver tumors although indications for ALPPS in children still need further research mainly focused on validation of the minimally needed FLR in children undergoing extended liver resections. To our knowledge, this is the youngest patient on whom ALPPS was performed, and the only one with a rhabdoid tumor.

## 1. Introduction

Surgical resection remains an important treatment of choice for a large number of primary and secondary liver tumors in children. Sometimes, if a tumor infiltrates a large part of the liver, the amount of liver tissue that would remain after surgical resection, named future liver remnant (FLR) is not enough for normal liver function and can lead to postoperative liver failure (PLF).

For patients whose liver is healthy, FLR must be 25–30% of total liver volume (TLV), measured with the help of computed tomography (CT) or magnetic resonance imaging (MRI) diagnostics. For patients planning to go through preoperative chemotherapy treatment, FLR must be around 40% or above [1].

The initial treatment of choice would be a portal vein embolization to stimulate liver hypertrophy, which normally requires 4–8 weeks before final treatment in terms of hepatectomy. Sometimes the time needed for liver hypertrophy results in additional tumor growth and infiltration of major structures, thus leading to the fact that complete resection of the tumor is not possible.

Associating liver partition and portal vein ligation for staged hepatectomy (ALPPS) was first described in a publication by the liver surgery group led by Dr. Hans Schlitt in Regensburg, Germany, in 2012 [2].

ALPPS is a two-stage procedure. The first stage consists of portal vein ligation and liver tissue resection of the segments that are affected by tumor tissue while preserving hepatic arterial and venous flow and bile ducts. The second stage is the final treatment in terms of hepatectomy. The procedure provides sufficient liver hypertrophy for a second-stage hepatectomy in a much shorter time span than conventional methods.

By adding in situ splitting of the liver tissue to portal vein ligation during the first stage of the procedure, more rapid FLR hypertrophy is induced, thus allowing a safer hepatic resection (second stage) within a shorter period of time (3 to 14 days) compared to 4 to 6 weeks with the conventional two-stage procedure [1].

The aim of this paper is to present the first case of a pediatric patient with a marginally resectable rhabdoid tumor of the liver, previously considered to be unresectable, treated with a two-stage hepatectomy. To our knowledge, this is the youngest patient with this type of tumor that underwent a full ALPPS procedure.

## 2. Case Report

A two-month-old female infant was admitted to the hospital with a two-week history of abdominal distension, vomiting and lack of stool, initially treated with antibiotics and enema at a local hospital. Upon arrival, the patient was admitted to the intensive care unit (ICU) in stable condition but tachycardic and tachypneic with a distended abdomen. Physical examination revealed a large abdominal tumor palpable 5 cm below the costal margin, extending to the midline of the abdomen. Her body weight and height were 6200 g and 67 cm, respectively.

Initial laboratory workup included liver function test exam, lactate dehydrogenase (LDH), and alpha-fetoprotein (AFP) level (international normalized ratio (INR): 1.38, albumin: 24.52 g/L, aspartate aminotransferase (AST): 70.0 IU/L, alanine transaminase (ALT): 55.0 IU/L, total bilirubin: 52.63 mmol/L, LDH: 243.60 IU/L, AFP: 162.8 mg/L). Hepatitis virus serology was negative.

**Figure 1:**
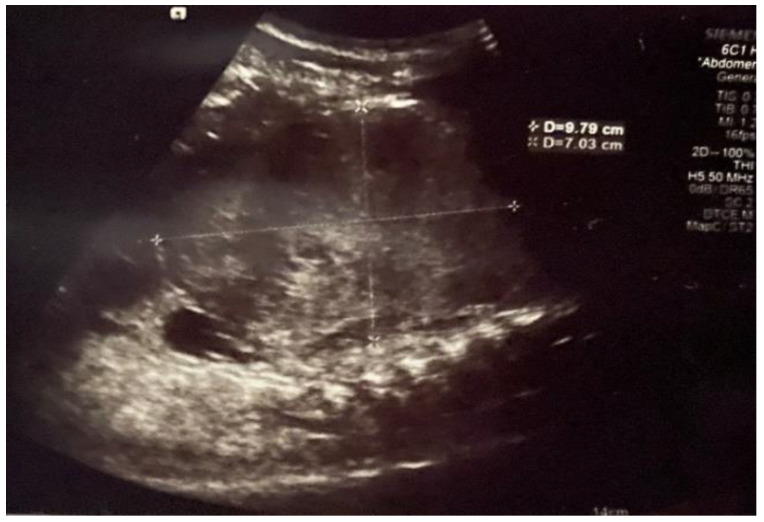
Initial abdominal ultrasound showing a large heterogeneous liver mass.

Initial abdominal ultrasound (US) revealed a large abdominal mass occupying the upper and middle aspect of the abdomen, probably of liver origin (Figure 1).

**Figure 2:**
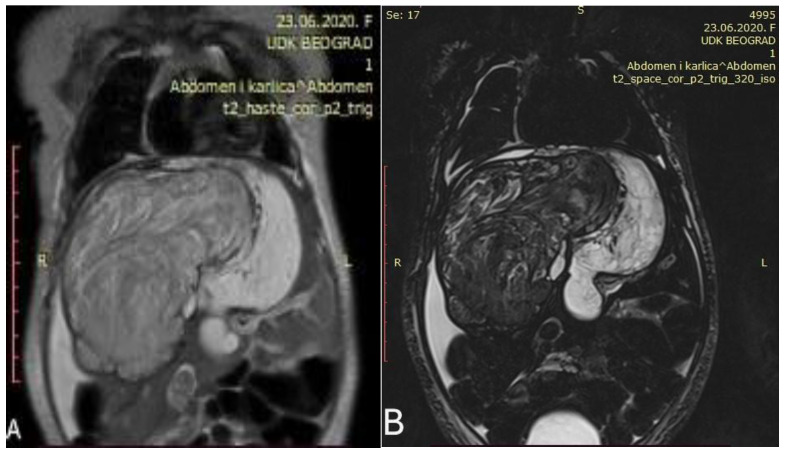
Initial abdominal MRI showed a voluminous heterogeneous tumor occupying the right liver ((**A**,**B**) Different coronal slices of the same MRI).

Abdominal MRI revealed a large heterogeneous liver mass that infiltrates most of the right liver (except Couinaud segment VI and VII) and almost the entire left lobe, measuring 120 mm × 85 mm × 78 mm with signs of parenchymal bleeding and necrosis (Figure 2).

A surgical biopsy of the tumor was performed, and pathohistological (PH) analysis showed an undifferentiated small round cell tumor or hepatic malignant rhabdoid tumor. The immunohistochemical study revealed positivity for vimentin, CK (AE1/3), CD8/18, CK19, EpCam (MOC31), CD 99 and anti-EMA (Epithelial Membrane Antigen) antibody, which diffusely marked the cellular cytoplasm.

The patient was started on chemotherapy composed of Vincristine, Doxorubicin and Cyclophosphamide according to the European Rhabdoid Registry (V6.2021) with the intention of reducing tumor size before possible resection. After six cycles of chemotherapy at the age of four months, the control abdominal ultrasound showed a significant reduction in the size of the tumor.

**Figure 3:**
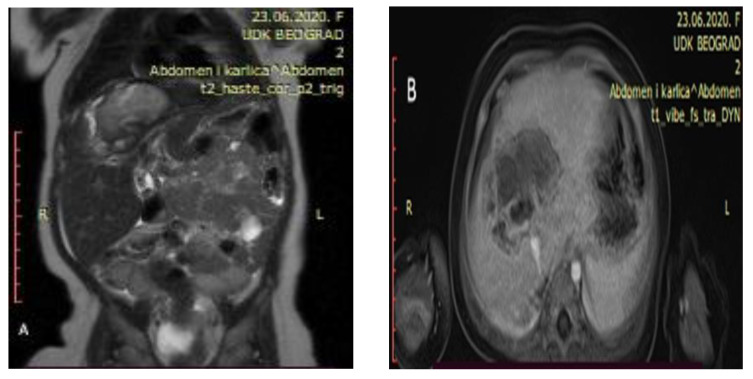
Control abdominal MRI after six cycles of chemotherapy showing a significant decrease in the tumor mass ((**A**) showing the coronal plane view of the MRI; (**B**) showing the axial plane view of the MRI).

Control abdominal MRI showed a significant decrease in the initial tumor mass (62 mm × 47 mm × 45 mm) with mostly cystic and necrotic morphology (Figure 3). The right hepatic vein was intact, the intermediary hepatic vein went straight through the tumor, and the left hepatic vein encircled the tumor next to its border. Both portal vein branches were intact as well as the inferior vena cava and hepatic arteries.

**Figure 4:**
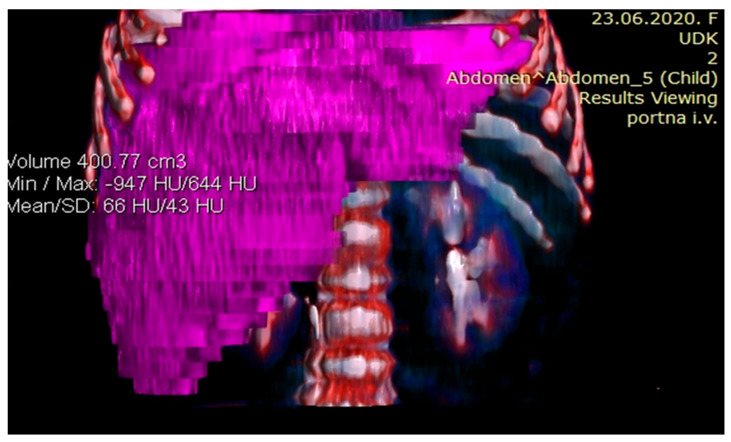
Preoperative volumetry of the liver showing total liver volume (TLV).

**Figure 5:**
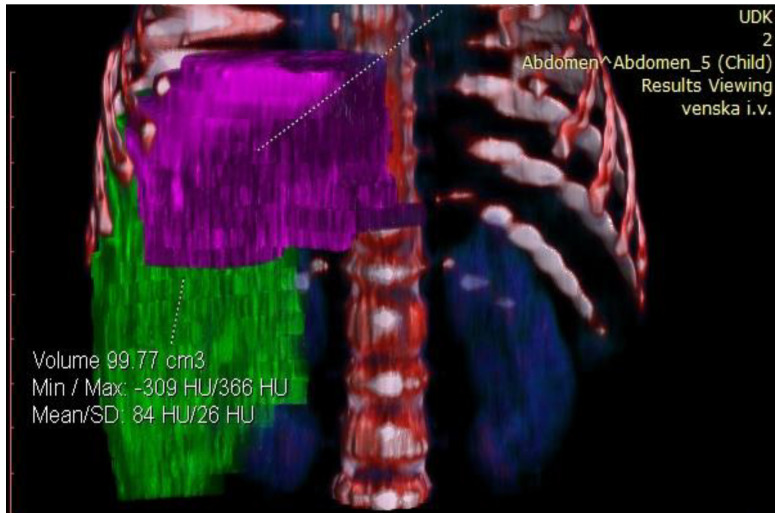
Preoperative volumetry of the liver showing future liver remnant (FLR).

Considering the tumor’s size and localization, volumetric measurement was indicated to assess its resectability. Volumetric assessment of the liver showed that FLR was 99.77 cm^3^. The estimated total liver volume (ETLV) was 400.77 cm^3^, with an FLR/ETLV ratio of 24%, which was considered small for size after major hepatectomy and would lead to PLF (Figure 4 and Figure 5).

According to those results, it was decided to proceed with the ALPPS procedure.

**Figure 6:**
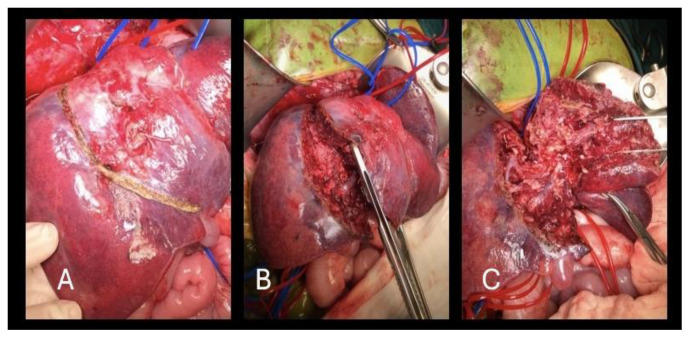
ALPPS (stage I)—(**A**) marked line for tissue in situ splitting dividing tumor tissue from healthy liver; (**B**) advanced phase of in situ splitting; (**C**) final phase of dividing tumor tissue from healthy liver.

Intraoperative finding was a large mass in the liver parenchyma that infiltrated segments IVa, IVb, V and VIII with an unclear border against surrounding tissue. After identification of the portal structures, the left portal vein was marked and transected. The left hepatic artery and right hepatic vessels were safeguarded, and tumor tissue was completely physically separated from the surrounding liver parenchyma (segments VI and VII) and placed in a plastic bag (Figure 6). The parenchymal split was performed by Cavitron ultrasonic surgical aspirator (CUSA—Valleylab; Boulder, CO, USA).

**Figure 7:**
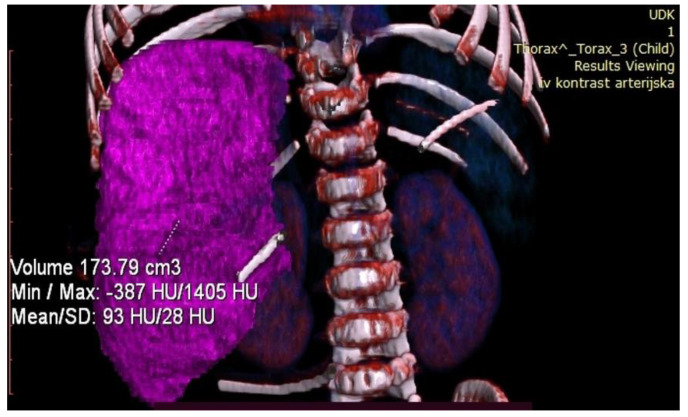
Postoperative volumetry of the liver showing future liver remnant.

Control volumetry performed on the seventh postoperative day showed that the FLR was 173.79 cm^3^, which represented a 74.1% gain in volume, and a FLR/ETLV ratio of 43% (Figure 7).

**Figure 8:**
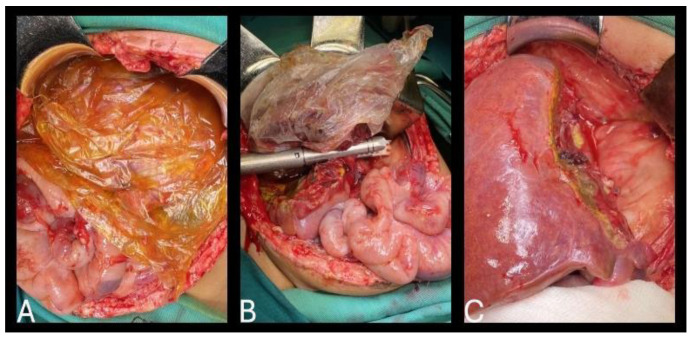
ALPPS procedure (stage II)—(**A**) tumor tissue isolated in a plastic bag prior to second stage; (**B**) dividing remaining vascular structures; (**C**) remaining healthy liver without tumor tissue.

The second stage of the ALPPS procedure in which the tumor was completely removed after division of the left hepatic artery, left biliary pedicle, left and middle hepatic vein was performed on the eighth postoperative day (Figure 8). Postoperative recovery was uneventful.

Histological examination confirmed the initial diagnosis of a malignant rhabdoid tumor with a clear resection margin. Control abdominal ultrasound showed regular blood flow through the right portal vein, right hepatic artery vein as well as inferior vena cava.

The patient was discharged from the hospital 15 days after the first operation.

Control US and CT follow-up examinations after one, three and six months revealed no signs of tumor recurrence. Unfortunately, after this period the patient no longer attended follow-up appointments.

## 3. Discussion

A large number of pediatric liver tumors at the time of diagnosis are unresectable and liver transplantation is considered as a treatment of choice because of insufficient FLR, which can lead to postoperative liver failure (PLF). The possibility of a complete tumor resection is in most cases determined by the characteristics of the tumor and a preoperative liver function status. Makuuchi et al. proposed preoperative vein embolization (PVE) in order to prevent PLF by enlarging the FLR volume [3]. From that moment, PVE or portal vein ligation (PVL) has been considered a standard treatment modality in treating patients with insufficient FLR. According to a large meta-analysis of 37 studies on 1088 patients, PVE takes 2–6 weeks to achieve a sufficient FLR with a mean volumetric increment by 8–27% volume, which is a major drawback of this procedure because the risk of tumor progression during this waiting period remains substantial and prevents an (R0) resection in 70% to 100% cases [4]. Alternatively, PVL achieved a FLR volumetric increment of 38–53% over a period of 4–8 weeks [5,6].

The ALPPS procedure was performed for the first time in 2007 by Professor Hans Schlitt from Regensburg, Germany.

In 2012, Schnitzbauer et al. introduced combining right portal vein ligation with splitting liver parenchyma in situ in 25 patients who were diagnosed with malignant liver tumors [2]. That procedure was eventually named ALPPS by de Santibanes and Clavien, who showed that significant augmentation of FLR could be achieved within one week, decreasing the risk of tumor progression [7]. In 2017, Hong et al. reported their experience of partial liver splitting at the first stage of ALPPS in children. The paper shows the feasibility and safety of performing a modified ALPPS procedure in very small infants (aged 54 days) with insufficient FLR [8].

The first series of pediatric patients that were diagnosed with initially unresectable liver tumors and were treated with ALPPS procedure was reported by JC Wiederkehr et al. in 2015. The authors noticed a rapid growth of the liver remnant in patients from their series. The increase in the FLR to TLV ratio ranged from 62 to 102% in all but one patient during the period of 7–12 days [9].

Total tumor resection in patients with malignant liver tumors is the only treatment model for them to achieve long-term survival. However, some tumor sizes or proximity to central structures leave insufficient liver tissue after surgical resection for the organ to maintain normal function.

Preoperative CT/MRI and staging of the tumor determine its resectability, after which an assessment is made as to whether FLR is going to be enough for the liver to function properly after surgical resection. Patients with a future liver remnant volume (FLR-V) of less than 25% are most likely to benefit from the ALPPS procedure. This 25% threshold, primarily based on adult patient data, forms the most compelling argument for utilizing the ALPPS procedure in pediatric patients, despite the lack of information in this group [10].

The main advantage of the ALPPS procedure is rapid FLR hypertrophy. It is thought that in situ parenchymal resection stimulates faster growth of the liver tissue when compared to similar procedures such as PVE, which does not include parenchymal resection. Children are well known for their rapid liver regeneration of the FLR, which can be as low as 20–25%. Because children tolerate major liver resections better than adults, the ALPPS procedure should only rarely be indicated for treating liver tumors in children [9].

Other liver surgery techniques also need to be considered, such as portal vein embolization (PVE), portal vein ligation (PVL), two-stage hepatectomy (TSH), and one-stage hepatectomy (OSH). ALPPS certainly does not replace previously mentioned options but may allow tumor resection in selected patients without any other surgical options left. Indications for choosing the ALPPS procedure as a treatment of choice must be clear [8,11,12].

The majority of patients are ones with colorectal liver metastases (CLRMs), hepatocellular carcinoma (HCC), and perihilar cholangiocarcinoma (PHC). Most of the cases in the pediatric population are patients with hepatoblastoma.

The ALPPS procedure should not be considered as a replacement for already existing surgical techniques in treating liver tumors but as an addition to a wide spectrum of techniques in the experienced liver surgeon’s hand. It should, at the moment, be considered a last resort when other surgical options are inadequate. From that perspective, it can certainly be observed as beneficial. Inappropriate patient selection in the future can make it detrimental, as is true for any other surgical procedure.

In every surgery, an indication of when to use it remains an essential decision, which is particularly true for the ALPPS procedure. Other surgical techniques, such as PVE or standard two-stage hepatectomy (TSH), are certainly not replaced by this procedure, but it can provide total tumor resection in patients without other surgical options left [13]. Surgeons should consider the ALPPS procedure for potentially treating patients with advanced-stage liver tumors, who can undergo liver transplantation [14].

In our case, the decision to choose the ALPPS procedure as a treatment of choice was because of the small FLR and a great risk of PLF, as well as the lack of time for treatment due to the patient’s age and the fact that the tumor was of rhabdoid extremely malignant pathology.

## 4. Conclusions

Associating liver partition and portal vein ligation for staged hepatectomy (ALPPS) procedure has emerged as a new strategy to increase resectability of the large hepatic tumors in children despite the fact that validated criteria for minimal FLR in pediatric liver resection are lacking and so are clear indications for ALPPS. Regardless of that fact, ALPPS can be a valuable technique to achieve complete resection of pediatric liver tumors, although indications for ALPPS in children still need further research mainly focused on validation of the minimally needed FLR in children undergoing extended liver resections.

Our case shows the feasibility of performing this procedure in very young infants with FLR as small as 20–25%, and also rapid growth of the remnant liver during the one-week period.

To our knowledge, this is the youngest patient on whom ALPPS was performed, and the only one with a rhabdoid tumor presented in the available literature.

## Data Availability

Data are contained within the article.
